# Expanding the Reach of Monoclonal Antibodies: A Review of Synthetic Nucleic Acid Delivery in Immunotherapy

**DOI:** 10.3390/antib12030046

**Published:** 2023-07-06

**Authors:** Christopher Chung, Sagar B. Kudchodkar, Curtis N. Chung, Young K. Park, Ziyang Xu, Norbert Pardi, Mohamed Abdel-Mohsen, Kar Muthumani

**Affiliations:** 1 GeneOne Life Science, Inc., Seoul 04500, Republic of Korea; cchung@genels.us (C.C.); skudchodkar@genels.us (S.B.K.); curtiscurtis335@gmail.com (C.N.C.); park@genels.com (Y.K.P.); 2 Massachusetts General Hospital, Harvard University, Boston, MA 02114, USA; zixu@mgh.harvard.edu; 3 Department of Microbiology, Perelman School of Medicine, University of Pennsylvania, Philadelphia, PA 19104, USA; pnorbert@pennmedicine.upenn.edu; 4 The Wistar Institute, Philadelphia, PA 19104, USA; mmohsen@wistar.org

**Keywords:** monoclonal antibodies, immunotherapy, synthetic nucleic acids, therapeutic delivery, accessibility, utility, diseases, in vivo production, administration logistics, cost reduction, biopharmaceuticals

## Abstract

Harnessing the immune system to combat disease has revolutionized medical treatment. Monoclonal antibodies (mAbs), in particular, have emerged as important immunotherapeutic agents with clinical relevance in treating a wide range of diseases, including allergies, autoimmune diseases, neurodegenerative disorders, cancer, and infectious diseases. These mAbs are developed from naturally occurring antibodies and target specific epitopes of single molecules, minimizing off-target effects. Antibodies can also be designed to target particular pathogens or modulate immune function by activating or suppressing certain pathways. Despite their benefit for patients, the production and administration of monoclonal antibody therapeutics are laborious, costly, and time-consuming. Administration often requires inpatient stays and repeated dosing to maintain therapeutic levels, limiting their use in underserved populations and developing countries. Researchers are developing alternate methods to deliver monoclonal antibodies, including synthetic nucleic acid-based delivery, to overcome these limitations. These methods allow for in vivo production of monoclonal antibodies, which would significantly reduce costs and simplify administration logistics. This review explores new methods for monoclonal antibody delivery, including synthetic nucleic acids, and their potential to increase the accessibility and utility of life-saving treatments for several diseases.

## 1. Introduction

Passive antibody therapy is the treatment of disease using infusions of antibodies made in the laboratory or in animals. This type of therapy differs from active immunity, which occurs when the immune system becomes activated after exposure to vaccines, pathogens, or cancer cells and subsequently generates antibodies within the host. The origins of passive antibody therapy can be traced back to the early 1890s with the work of Behring and Kitasato [1,2,3]. They demonstrated that serum from animals immunized against tetanus toxin could provide protection in other animals against tetanus, which became known as serum therapy. Before using antibiotics, this serum therapy was the first-line treatment for various infectious diseases, despite its associated side effects such as hypersensitivity reactions, serum sickness, and the risk of transmitting blood-borne pathogens [1,4]. However, due to the toxicity of serum therapy and its involved method of administration, its popularity declined in favor of antimicrobials, particularly in the context of infectious diseases. Nevertheless, with the emergence of monoclonal antibodies (mAbs), antibody-based therapy has been revitalized [5,6,7].

## 2. Monoclonal Antibodies: From Hybridoma to Humanized Transgenic Mice

Typically, foreign agents elicit a polyclonal antibody response in organisms where several different B lymphocytes produce antibodies that target multiple linear and/or conformational epitopes of the foreign molecule. In contrast, mAbs target a single epitope or antigen and are made in the lab by isolating the unique B cell that produces that antibody. Kohler and Milstein first introduced the generation of mAbs in 1975 using the hybridoma technique, which laid the foundation for developing mAbs-based therapeutics and diagnostic tools [8]. Briefly, this method involves the immunization of animals to generate a humoral immune response, followed by isolating the B cells from the immunized animals, usually from their spleen. These B cells are fused to immortal B cell cancer cells, myeloma, to produce hybridoma cells that are selected under hypoxanthine–aminopterin–thymidine (HAT) media to eliminate non-fused cells. The hybridomas are then serially diluted and screened to isolate single clones that all produce the same antibody to the determined target. This technique enables the production of antibodies of a defined specificity in large quantities ex vivo. Alternatively, more efficient methods of identifying mAbs have been explored, including various in vitro display technologies, in vivo phage display, and flow cytometry-based B cell screening and sorting [9,10,11]. The formats of mAbs have been improved over the years, from murine, chimeric, humanized, to fully human mAbs with successively decreasing degrees of immunogenicity in recipients [12]. These technologies have included the grafting of variable domains, the grafting of complementary determining regions, and the use of humanized transgenic mice.

## 3. Challenges of Making Monoclonal Antibody Therapies More Accessible: Current Limitations and Future Directions

Although mAb therapies are promising for treating various diseases, their accessibility to larger patient populations remains limited due to several challenges. One major challenge is that the current methods of generating high quantities of clinical-grade mAbs are arduous and extremely costly. The large size of mAbs precludes chemical synthesis methods, and the need for proper glycosylation and folding makes lower organism expression platforms unsuitable for commercial mAb production. Therefore, mammalian expression systems are needed to produce biologically functional mAbs. However, using mammalian expression systems is complicated and expensive due to the slow cell growth and low product yield. The most common cell culture line used for current synthetic mAb production is Chinese hamster ovary (CHO) cells, where genes encoding the mAb of interest are introduced into an expression vector that is subsequently stably transfected into CHO cells. Expressed mAbs are secreted into the supernatant and captured through affinity chromatography. However, the transfection, screening, and amplification processes using these cells are technically and logistically laborious, leading to increased production costs and making mAbs an expensive form of therapy [13].

Moreover, harsh conditions are often required to remove biological contaminants introduced by the expression system, which can result in product degradation, aggregation, and yield loss. Following manufacturing, careful formulation is required according to the solubility and intrinsic stability of the mAb product, further adding to the complexity of mAb therapeutics [14]. These challenges must be addressed before mAbs can be deployed on a wider scale and made more accessible to patients.

Recombinant mAbs are expensive therapeutics. For example, a full course of Campath^®^ (Alemtuzumab), a mAb used for treating multiple sclerosis, can cost upwards of USD 60,000 [15]. In contrast, Keytruda^®^ (Pembrolizumab), a Programmed cell death protein 1 (PD-1) inhibitor for treating various cancers, costs USD 10,897 for each dose every three weeks [16]. Additionally, most therapeutic mAbs are administered intravenously in a hospital setting, which can burden the healthcare system. To maintain efficacy, repeated administrations of these antibodies are typically required, and these therapies are frequently cost-prohibitive to patients. Although subcutaneous delivery of mAbs may address some of these shortcomings, not all mAbs can be formulated for this delivery method, as this approach is highly dependent on mAb solubility, viscosity, self-association, intrinsic stability, aggregation, and precipitation profiles [17]. All these barriers potentially limit the accessibility of these therapies to patients.

## 4. Approaches for Monoclonal Antibody Delivery: A Promising Alternative for Increased Accessibility and Efficiency

To overcome these challenges, gene therapy approaches have gained interest as an alternative method to produce mAb therapeutics. These methods involve delivering genes expressing mAbs of interest instead of the mAbs themselves, allowing in vivo mAb production in the body’s cells and eliminating the need for the production and purification processes currently used for mAbs, as mentioned above. The use of these gene therapy approaches for mAb administration can significantly reduce the costs of these treatments for several reasons. The manufacturing and purification of the viral vectors, DNA plasmids, or lipid nanoparticle-encapsulated mRNAs used to deliver the mAb genes can all be achieved more rapidly and with higher yields than protein-based mAbs. Gene therapy delivery platforms can also be administered easily into muscle or other tissues such as lung, spleen, lymph nodes, and bone marrow, reducing or eliminating the need for hospitalization [18]. Furthermore, depending on the platform, mAb genes may be expressed for several days or months, reducing the need for repeated delivery. In this review, we explore these new monoclonal antibody delivery methods, which can potentially increase the accessibility and utility of these life-saving treatments for several diseases.

## 5. Different Delivery Platforms for Monoclonal Antibodies

***(i) Adeno-associated virus***

Various methods are employed for gene delivery, including plasmid-coated gold particles, lipid–DNA complexes, naked DNA with electroporation, and viral vector-based delivery (Table 1). Among these, virus-based vectors are extensively researched, particularly adeno-associated virus (AAV) vectors. AAV is a small 25 nm non-enveloped virus that can infect both dividing and quiescent cells without integrating into the host cell genome. Due to its ability to enter target cells, transfer its genome to the nucleus, and maintain long-term expression, AAV is an attractive vector for gene therapy [19]. AAV-mediated gene delivery has shown a slow but persistent expression profile for several years compared to only several weeks with other viral vectors [20]. AAV also generates fewer adverse immune responses than other virus-based approaches because of its low uptake by antigen-presenting cells and limited presentation on major histocompatibility (MHC) complexes. This limits the host immune responses to both the vector itself as well as the transgene it is expressing [19].

Despite the advantages, AAV delivery has some limitations, such as the capacity to encapsulate only up to 3.3 kb of DNA, limiting the types of mAbs that can be delivered using this method. The use of alternative capsids and self-complementary AAV (scAAV) vectors, though increasing expression, further limits carrying capacity [21,22]. Additionally, pre-existing or host-generated anti-AAV immune responses can neutralize the virus, reducing its efficacy in repeat dosing. This concern, however, appears to be more of an issue with the use of adenovirus-based delivery vectors [23]. Studies have shown that 96% of patients given AAV therapy develop antibodies to the virus, and 32% had neutralizing antibodies in vitro. These neutralizing antibodies can limit AAV-mediated transduction in organs such as the liver and lung, but no limits were seen on gene delivery to muscle, brain, and retina [19]. Additionally, in many instances, long-term expression of the gene is not required and may, in fact, be harmful. Limiting the length of expression of these genes could be important to prevent any off-target effects and prevent long-term complications.

AAV-mediated delivery of monoclonal antibodies (mAbs) has been shown to be effective in both prophylactic and therapeutic applications in a variety of diseases, including Ebola, malaria, influenza, HIV, cancer, Alzheimer’s, and drug abuse [24,25,26,27,28,29,30,31]. The feasibility of this approach for mAb therapy was first demonstrated by Lewis et al. in an HIV model, where a recombinant AAV vector was used to deliver human antibody IgG1b12 to Rag1-deficient mice. The mice expressed IgG1b12 from muscle with gp120-binding specificity, and the antibody was able to neutralize both T cell line-adapted and primary HIV-1 isolates. After a single intramuscular (IM) administration, the recombinant AAV (rAAV) genome persisted for over 6 months [32]. Since then, the concept of antibody gene transfer using AAV was adapted to macaques by Johnson et al., who generated Simian immunodeficiency virus (SIV)-specific immunoadhesins, antibody-like molecules that fused the Fc region of an immunoglobulin and the functional domain of a binding protein and packaged them into AAV1 capsids. A single intramuscular injection of the AAV vector resulted in long-term (>1 year) continuous expression of a biologically active protein. Six out of nine immunized monkeys were protected against infection by the SIV challenge, and all nine were protected from AIDS. In contrast, all six of the controls became infected, and two-thirds (four of six) died over the course of the experiment [6]. Balazs et al. also demonstrated that AAV delivery of full-length human broadly neutralizing antibodies into humanized mice provided complete or partial protection from CD4 depletion following HIV infection [7].

Preclinical studies in mice and sheep have shown that AAV-based mAbs have good safety and tolerability profiles. No significant changes in blood chemistry or hematological parameters were observed, and only mild myositis was reported at the injection site, with no observed toxicity in major organs [33]. Two phase 1 clinical trials have been conducted to evaluate the safety and tolerability of AAV-delivered mAbs: one using broadly neutralizing HIV mAbs PG9 in healthy male adults and another using VRC07 in HIV-1 infected adults [34,35]. In both studies, intramuscular AAV-mediated mAb delivery was safe and well tolerated. In the PG9 study, vectored immunoprophylaxis using AAV1 resulted in serum neutralization in four of the twenty-one volunteers. While muscle biopsy confirmed PG9 expression, its expression level was too low to detect in circulation, directly suggesting very low expression. In contrast, in the clinical trial using AAV8-delivered VRC07, measurable mAb was detected in all three dose groups, with maximal concentrations exceeding 1 µg/mL in three individuals. Though target trough concentrations sufficient for protection have not been identified for VRC07, based on previous studies using the less potent mAb VRC01, exceeding 1 µg/mL with VRC07 is considered a reasonable therapeutic target [36]. Pseudoviral neutralization studies demonstrated the functionality of in vivo-generated VRC07 [35].

Although AAV-based mAb delivery has shown promise, several challenges still need to be addressed, some of which are common to other nucleic acid-based delivery methods. Immune responses to mAbs are a significant obstacle that needs to be overcome [6,37]. The formation of antibodies against the mAb of interest is known as the anti-drug antibody (ADA) response. Studies have shown that both heavy and light chains are targeted, mainly or exclusively, to variable regions, and reactivity to complementarity-determining region (CDR)-H3 peptide has been demonstrated. The magnitude of anti-antibody responses highly correlates with the degree of sequence divergence of the delivered antibody from the germline [38]. However, in some cases, ADA is not observed. For example, in an SIV challenge model study, one macaque maintained 240–350 μg/mL of anti-SIV antibody 5L7 for over six years with little to no anti-drug antibodies and remained uninfected [24]. Various strategies are being explored to overcome this challenge.

For instance, the liver is an immunologically tolerant organ; therefore, liver-directed expression may help limit ADAs [39]. Resident antigen-presenting cells such as dendritic cells, Kupffer cells, liver sinusoidal endothelial cells, hepatocytes, and hepatic stellate cells present antigens in a tolerogenic manner to T cells and express immunosuppressive cytokines, resulting in regulatory T cells (Tregs) expansion, effector T cell anergy or death, and type 1 regulatory cell induction [40]. Likewise, AAV-mediated gene therapy targeting the liver has been shown to induce antigen-specific Tregs [41,42,43,44,45]. A rhesus macaque study observed no ADA to 4L6 mAb when the AAV8 vector was administered intravenously using a liver-specific TBG promoter. Priming with AAV8-CMV or AAV8-TBG, followed by boosting with AAV1-CMV, significantly increased 4L6-IgG1, accompanied by a weak ADA response [46]. Although it has a relatively low immunogenicity profile compared to other viral vectors, AAV can still give rise to immune responses, making anti-vector immunity another hurdle. Cellular immune responses against AAV capsid limit transgene expression by eliminating transduced cells [47]. Notably, a non-human primate study has shown that the elicitation of capsid-specific CTLs is limited to AAV capsids that exhibit heparin-binding activity. Serotypes like AAV8, which lack heparin-binding activity, did not induce CTL responses [48]. Vector administration leads to seroconversion [49]. Humoral immune responses can generate AAV capsid-neutralizing antibodies, which block transduction and prevent re-administration of that particular gene therapy vector [50]. Transgene insertional mutagenesis risks also remain a concern. AAV integration has been shown to cause hepatocellular carcinoma (HCC) in mice. In dogs given AAV gene therapy for hemophilia, integration events were noted in or near genes associated with cell growth [51,52]. Identifying AAV genomes in tumor-associated genes of HCCs in patients raised safety concerns about the therapeutic use of AAVs [53]. However, whether AAV integrates into oncogenes in humans is not known and the risk thus far remains theoretical.

***(ii) Plasmid DNA-based monoclonal antibody delivery***

Given the challenges associated with viral vector-based synthetic mAb delivery approaches, researchers have explored alternative approaches, such as the use of “naked” DNA plasmids to deliver the necessary genes for in vivo production of immunotherapeutic mAbs. DNA-based mAb delivery originated from observations made during investigations of DNA vaccines. For example, in 1990, Wolff et al. reported protein expression from unformulated, naked DNA expression vectors injected into mouse skeletal muscle in vivo [5]. This observation led to the investigation of a DNA vaccine platform [54,55]. Although, at the same time, administration of DNA with a needle and syringe gave good expression in animal models, first-generation DNA vaccines induced weak cellular responses and weak or nonexistent antibody responses in many clinical studies [56,57,58,59,60,61,62].

Over the years, several strategies were discovered to enhance the expression of plasmid constructs. In the early 2000s, it was found that the delivery protocols incorporating “adaptive” in vivo electroporation (EP) can promote greater gene delivery into cells, leading to increased expression of the construct. This physical process exposes cells to a brief electrical field pulse that induces temporary pores in the cell membrane and promotes DNA electrophoresis, thus aiding DNA uptake. This technology was then applied to the in vivo production of mAbs in 2004 with the work of Tjelle et al. and Perez et al. [63,64]. EP would later be further refined through pulse pattern, array spacing, and voltage optimizations. Other methods of increasing expression, such as codon/RNA optimization to improve protein translation and RNA stability, were then established [65,66]. Additionally, leader sequences were added to genes to enhance translational efficiency, while strong promoters were used to drive expression.

Several advances were made to the delivery to improve in vivo expression of DNA-encoded mAbs (Table 1). Hyaluronidase treatment, which helps DNA move through the extracellular matrix and enhance plasmid uptake, would be administered either before DNA delivery or co-formulated with DNA [67,68]. Modifying the antibody to resemble the human parental germline antibody sequence would also increase overall production in vivo while preserving functionality [68,69]. Mutations such as triple Fc modification M252Y/S254T/T256E were introduced to promote neonatal Fc receptor-mediated recycling of IgG into circulation, thereby extending mAb half-life [70]. Incorporating these additional refinements would allow for in vivo mAb expression at biologically relevant levels in small animal models. The potential value of the DNA-based mAb delivery platform has thus far been demonstrated in many models of infectious diseases such as Ebola, *Pseudomonas aeruginosa*, influenza, chikungunya, dengue, Zika, and HIV, as well as cancer types including breast, ovarian, and prostate [68,71,72,73,74,75,76,77,78,79]. The equivalency of binding for in vivo delivered DNA-based mAbs to recombinant mAbs has been shown through epitope mapping [68]. In addition to their use alone, DNA-based mAbs can be combined with protective vaccines, providing both immediate and persistent protection and establishing vaccine-induced memory responses [74].

Non-viral DNA delivery offers several advantages over AAV-based antibody delivery, including increased gene insert size, avoiding immune responses directed against the AAV vector and thus avoiding host seroconversion, enabling repeat delivery, and ease of manufacture and manipulation. In addition, DNA is less immunogenic, and the temporal nature of the delivered DNA plasmid means there is no long-term risk from the delivery vehicle. Furthermore, the DNA-plasmid approach is highly cost-effective, as it can be produced in large quantities using established bacterial fermentation processes. Lastly, DNA is more stable at room temperature than proteins, viral vectors, and LNP-encapsulated mRNAs, further reducing costs by minimizing the need for cold chains during storage and transport. These advantages make DNA-delivered mAbs a more broadly applicable alternative to vector-based or traditional recombinant purified monoclonal antibodies.

DNA-based mAb delivery platforms offer several advantages but have some limitations; for instance, peak expression takes approximately 7–14 days, which may be too delayed for therapeutic purposes. In addition, EP devices are not standard equipment in most hospitals, so additional expenditure and training would be necessary to deploy this system. Even then, electroporation devices required for delivery may not be suitable for clinical settings due to their potential to cause muscle contractions, pain, and tissue damage [80,81]. Alternative delivery methods such as hydrodynamics injection and suction-based transfection are being investigated for DNA vaccines and may apply to DNA-encoded mAbs [82,83]. Though not seen in the clinic, theoretical risks of insertional mutagenesis and induction of autoimmune antibodies against DNA from repeat injections exist. As with AAV, anti-drug antibodies remain an issue, although redosing is possible as there is no vector immunity [71,73,76,84]. Transient T cell depletion has been used in immunocompetent mice to prevent animals from developing anti-antibody responses, enabling long-term expression. However, this may not be a translatable clinical strategy [68,76]. Two phase 1 clinical trials have been conducted for DNA-based mAbs using EP and hyaluronidase, one for the Zika virus (using INO-A002) and the other for preventing COVID-19. The INO-A002 study has been completed, but no results are available yet, while the COVID-19 study is actively recruiting.

***(iii) mRNA-based mAb delivery***

**Figure 1:**
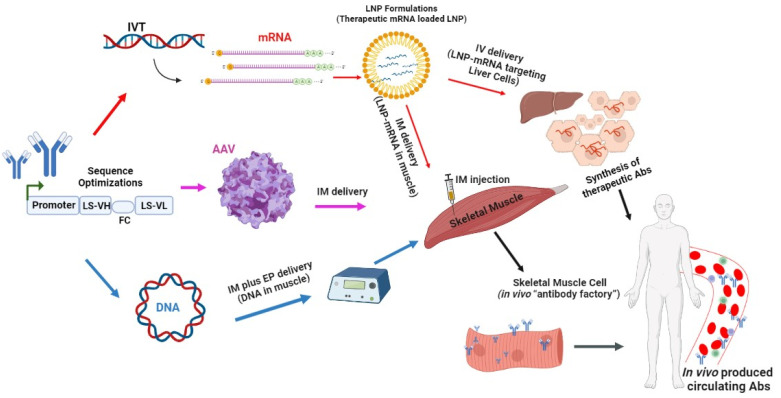
An overview of mRNA-based delivery of mAb.

Advances in synthesis, purification, and delivery methods have renewed interest in messenger RNA (mRNA)-based delivery approaches. mRNA-based delivery of mAbs share many of the benefits of DNA-based approaches but have the advantage of faster protein expression (within hours), greater peak expression without mutational risks, and no anti-vector immunity allowing for repeated dosing [85,86,87,88,89]. Moreover, the short-lived nature of mRNA allows for controlled expression and minimizes the risk of multisystem inflammatory syndromes or other side effects associated with introducing foreign agents [90,91] (Figure 1).

The manufacturing process for mRNA-encoded mAbs is highly efficient. Within weeks, clinical batches can be produced after obtaining the sequence encoding the mAbs of interest, and the process is scalable and cell-free. A facility dedicated to mRNA production could potentially manufacture mRNA-encoded mAbs against multiple targets with minimal adaptations to process and formulation [92,93,94].

The delivery of mRNA to the cytosol leads to the synthesis of the encoded mAbs, which is then subjected to post-translational modifications, resulting in a fully functional product delivered to the correct cellular compartments for proper function, usually within hours. The peak expression of mRNA-encoded antibodies typically occurs within days [95,96,97,98,99,100]. The genetic material carried by mRNA is expressed transiently until the mRNA is degraded. The durability of protein expression can range from hours to days depending on the dose of mRNA, the properties of the mRNA (optimizations), and the route of mRNA delivery [101]. The stability of mRNAs depends on various factors, including the 5′ cap, poly(A) tail, and various cis-elements, such as linear A + U- and C + U-rich motifs and stem-and-loop structures, which interact with trans-acting factors affecting the rate of decay [102]. The 5′ and 3′ UTRs that recruit RNA-binding proteins and microRNAs are crucial for stability and translation [103]. Additionally, circular RNAs can increase the half-life of protein production up to threefold compared to linear mRNA in vitro [104]. Like other nucleic acid-based approaches, mRNA can encode multiple proteins with different chemical and physical properties without major changes in their physiochemical properties, thus allowing for simple and cost-effective manufacturing.

The mRNA templates or byproducts from in vitro transcription can activate the innate immune system via recognition by pattern recognition receptors (PRRs), decreasing protein expression, reducing the mAbs’ longevity, and inducing adverse events in the host. Toll-like receptors 3, 7, and 8, retinoic acid-inducible gene 1 (RIG-I), and nucleotide-binding and oligomerization domain-containing protein 2 (NOD-2) are PRRs that recognize mRNA and its contaminants, leading to the production of proinflammatory cytokines and type I interferons [105,106]. To decrease the immunogenicity of mRNA, base modifications like pseudouridine or 5-methylcytidine, which are naturally occurring in RNA, can be incorporated [107,108,109,110]. Enriched GC content can also decrease immune responses while increasing expression several folds higher [111]. The immunogenicity of the platform can be further reduced by removing contaminants like short RNAs and double-stranded RNAs from the in vitro transcription process using techniques like high-performance liquid chromatography, anion exchange chromatography, affinity chromatography, size exclusion columns, cellulose purification, or RNase III treatment [86,87,88,112,113].

Due to its inherent instability, mRNA-encoding monoclonal antibodies require carrier assistance or delivery platforms to protect against nucleases and ensure effective delivery. Various strategies have been developed, including lipid-based, polymer-based, peptide-based, virus-like replicon particle, cationic nano-emulsion delivery, and direct injection into cells. Lipid nanoparticles (LNPs) are currently the most versatile and potent delivery system [101]. These nanoparticles are composed of ionizable amino lipids, polyethylene glycol, phospholipids, and cholesterol and are negatively charged nucleic acid delivery platforms [114]. The ionizable amino lipids facilitate self-assembly, cellular uptake of mRNA, and escape from the endosome by interacting with the endosomal membrane. Polyethylene glycol prolongs circulation time by preventing the binding of mRNA and plasma proteins, which would otherwise lead to clearance by the reticuloendothelial system. Phospholipids support the structure of the lipid bilayer, while cholesterol stabilizes the lipid nanoparticle structure [115]. These lipid nanoparticles provide two key advantages as a delivery platform for mRNA. Firstly, they protect mRNA from degradation by endosomal enzymes. Secondly, they can take advantage of existing cellular pathways to enhance mRNA expression. The first mechanism involves utilizing the Apolipoprotein E (ApoE)–Low-Density Lipoprotein Receptor (LDLR) pathway, a highly efficient delivery system [116]. Subsequently, the lipid nanoparticles are taken up through TLR4-mediated endocytosis, forming a vesicle that fuses with endosomes. Following this, the LNPs escape from endosomes, releasing mRNA into the cytoplasm to initiate protein synthesis.

LNPs are a popular delivery platform for mRNA due to their ability to protect mRNA from degradation and promote mRNA expression through endogenous cellular pathways. However, LNPs also have inherent immunogenicity that can lead to immune responses, which may not be desirable for in vivo expression of mAbs [117,118,119]. The size, surface charge, and repeat dosing of LNPs have all been shown to affect their immunogenicity [120,121,122]. Repeat dosing can also carry the risk of acute immune toxicity, known as complement activation-related pseudoallergy (CARPA), and can lead to the production of anti-polyethyleneglycol (PEG) antibodies, which are associated with both anaphylactic responses and accelerated blood clearance [123,124]. Cellular toxicities may also occur through the accumulation of lipids. These effects can be alleviated through the optimal formulation of mRNA and LNP. Aside from optimizing vesicle size and surface charge, other methodologies that have been explored include the incorporation of aliphatic ester prodrugs of anti-inflammatory steroids and the substitution of less immunogenic polymers [125]. Notably, CARPA and serious adverse events were not observed after the second dosing in non-human primate preclinical studies and a clinical trial assessing mRNA-encoded Chikungunya virus antibody [95,126].

Compared to viral vector- or DNA-based approaches, mRNA-based methods offer a faster route to protein production since the mRNA does not require nuclear localization to produce a functional protein. In a study by Pardi et al., VRC01 antibody expression was detected within hours and peaked at 24 h after IV injection of LNP-formulated m1Ψ-modified mRNA encoding the heavy and light chains of the antibody in BALB/c mice [97]. However, other studies have reported peak expression not occurring until seven days post-administration [127]. These expressed mRNA-delivered mAbs can then persist in the body for several months after administration [126,128]. Furthermore, mRNA-based approaches may potentially avoid antibody production against the encoded protein. Pardi et al. found no anti-EPO antibody production in BALB/c mice that received weekly intraperitoneal injections of 0.1 μg of murine erythropoietin (muEPO) encoding mRNA over five weeks [97] (Table 1).

One significant drawback of mRNA-encoded mAbs is that their administration is primarily limited to the intravenous (IV) route, with the liver being the target organ. This mode of administration is time-consuming and expensive, as mentioned earlier. However, Erasmus et al. demonstrated a novel approach that makes the intramuscular route a viable option, despite its limited number of target cells [129]. They combined self-amplification of an mRNA message encoding the antibody sequence with the co-expression of viral genes that antagonize the host’s innate immune response using alphavirus replicons. In their construct, alphavirus-derived self-amplifying replicon RNA (repRNA) maximized protein secretion on a per-cell basis. Serum concentrations of mRNA-expressed Zika virus (ZIKV)-117 antibody were over 30-fold higher than those of pseudouridine-modified or unmodified non-replicating mRNAs. Although self-amplifying mRNA may have also allowed for longer expression, this study did not investigate time points beyond 10 days. The high levels of ZIKV-117 mAb expression led to protection against lethal ZIKV infection in mice [129]. Despite the mAb levels being adequate for providing protection, it should be noted that double-stranded RNA intermediates formed during amplification may activate innate immunity, and the presence of replicon genes can also be immunogenic as well as lead to size constraints for the mAb gene [130,131,132].

Vanover et al. investigated an alternative administration route for mRNA-encoded mAbs in polyplexes by utilizing nebulized delivery of a glycophosphatidylinositol (GPI)-linked mRNA encoding a neutralizing mAb against SARS-CoV-2 infection [133]. The GPI-linked mAbs were anchored to the plasma membrane and induced retention in the lung. This approach increased the mAb half-life in lung tissue from 1.3 to 7.1 days. The mRNA-encoded mAbs (COV2–2832 or DH1041) prevented infection in a hamster SARS-CoV-2 prophylactic challenge model. In this study, mRNA-delivered mAbs protected hamsters from weight loss while reducing lung viral titers and loads. Viral nucleocapsid RNA detected by RNAscope, a commercially available in situ hybridization assay for detecting RNA in formalin-fixed paraffin-embedded tissue, showed decreased signal in the alveolar space but little effect on larger airways. However, reduced staining for SARS-CoV-2 spike protein was observed in both the airways and alveolar spaces. In addition, mRNA-treated hamsters had significantly lower lung and airway pathology scores. These findings indicate that localized delivery of mRNA-expressed mAbs can be highly effective. Furthermore, targeted delivery of mAbs to their site of action is favorable as it allows for lower doses of formulated mRNA to be utilized.

To date, only one clinical trial (led by Moderna) has investigated mRNA-encoded mAbs [126]. This trial involved the administration of mRNA-1944, which encodes the heavy and light chains of a Chikungunya virus (CHIKV)-specific monoclonal neutralizing antibody, CHKV-24, to healthy participants aged 18–50 years. Across doses, adverse effects were mild to moderate in severity and did not worsen with the addition of a second dose. At 12, 24, and 48 h after a single infusion of either 0.1, 0.3, or 0.6 mg/kg, dose-dependent levels of neutralizing CHKV-24-IgG were observed, which were predicted to be protective against CHIKV infection (≥1 µg/mL). These levels were sustained for ≥16 weeks at the 0.3 and 0.6 mg/kg doses, with a mean half-life of approximately 69 days. A second 0.3  mg/kg dose for the 0.3 mg/kg group administered one week after the first increased CHKV-24 IgG levels 1.8-fold. The ionizable lipid component of the lipid nanoparticle was readily eliminated, and CHKV-24 IgG mRNA was rapidly cleared within 48 h. Notably, no anti-PEG or anti-CHKV-24 IgG antibodies were detected in this study [126].

***(iv) Nanobodies***

Nanobodies are single-domain antibodies derived from camelids. Camelids produce antibodies that do not contain a light chain. The camelid antibody consists of two constant regions, a hinge region, and the antigen-binding domain variable heavy domain of heavy chain (VHH). Nanobodies are derived from the recombinant production of a VHH [134]. Nanobodies have binding affinities that are comparable to or better than conventional mAbs [135,136]. While the small size of nanobodies is advantageous for tissue penetration and passage through the blood–brain barrier, it also leads to rapid renal elimination and, therefore, a short serum half-life. Strategies to prolong the half-life of nanobodies include the addition of polyethylene glycol, albumin, or Fc fragment to the nanobody [137,138]. Rather than prolonging half-life, rapid clearance of nanobodies may be addressed by nucleic acid-based approaches which allow for long-term, stable production of nanobodies [139]. Like mAbs delivered in nucleic acid form, nanobodies delivered in this manner bypass in vitro production and characterization. Moreover, as before, AAV- or DNA-based delivery would avoid the need for repeated protein infusions and allow for stable concentrations of nanobody in vivo [140]. The delivery of nucleic acid-based nanobodies has also been explored in the context of circular RNAs, an area of increasing interest given their enhanced stability and more durable protein expression when compared to mRNA. Full-length mAb delivery using the circular RNA platform has yet to be reported, but Qu et al. demonstrated the feasibility of circular RNA therapeutics using a SARS-CoV-2 neutralizing nanobody [141]. In this study, supernatant of HEK293T cells transfected with the SARS-CoV-2 neutralizing nanobody circular RNA construct was able to effectively inhibit SARS-CoV-2 pseudovirus infection.

**Table 1:** A comprehensive review of monoclonal antibody delivery platforms.

AAV-Based Delivery				
Encoded mAb(s)	Modifications	Experimental Model	Mode of Delivery	Reference
anti-HIV-1 gp160 (IgG1b12)	dual promoter (pCMV/HC/EF1a/LC)	Rag1 mice	rAAV, IM	[32]
anti-human EGFR (14E1 and 14E1A, ablated CDR3)		nu/nu mice	AAV2/1, IM	[142]
anti-HIV gp41 (4E10) and anti-HIV gp120 (b12)		Rag2^−/−^γc^−/−^, NSG, B6, Balb/C	AAV2/8, IM	[7]
anti-Aβ		C57BL/6 mice	AAV1, IM	[29]
anti-ganglioside GM3(Neu5Gc) (14F7, mouse IgG1)		BALB/c mice	AAV2/9, IM, IV	[143]
anti-METH	scFv, self-complementary AAV	BALB/c mice	AAV8, IV	[30]
anti-HIV-1 gp41 (10E8), anti-HIV-1 gp120 (3BNC117, 10-1074)	“LS mutation” (M428L (Leucine)/N434S (Serine)) to increase half-life, vectors included specific miRNAs to promote transcriptional cleavage of transgene’s mRNA in APCs	rhesus macaques	AAV1, IM	[27]
anti-SIV gp120 and gp140 (5L7)		Mamu B*08-neg B*17-neg female Indian-origin rhesus macaque	AAV1, IM	[24]
anti-PD-1 (Nb11)	nanobody (VHH)	C57BL/6 mice	AAV8, IV	[28]
anti-EBOV GP2 internal fusion loop (CA45)	F129L, Y445F, and Y731F mutations in the AAV6 capsid	BALB/c mice	AAV6.2FF, IM	[25]
anti-MARV GP (MR78, MR82 and MR191)	bicistronic, CASI promoter	BALB/c mice, Dorset lambs	AAV6.2FF, IM	[144]
**DNA-Based Delivery**				
**Encoded mAb(s)**	**Modifications**	**Experimental Model**	**Mode of Delivery**	**Reference**
anti-human thyroglobulin	tet-off, tet-on	C57BL/6 mice, C3H mice	IM + EP	[63]
anti-I-Ed, anti-IgDa, anti-NIP		C57BL/6 mice, BALB/c mice, BALB.B mice, C.B-17 mice	IM + EP	[64]
anti-HIV Env (VRC01)		BALB/c mice	IM + EP	[65]
anti-DENV nAb	LALA mutation	Foxn1/NuJ mice	IM + EP	[72]
anti-CHIKV envelope		B6.Cg-Foxn1nu/J mice	IM + EP	[74]
anti-HER2	modification of VL sequence by replacing asparagine at amino acid 65 with serine to remove potential N-glycosylation site	BALB/c mice	IM + EP	[145]
anti-Influenza (A and B)		BALB/c and CAnN.Cg-Foxn1Nu	IM + EP with hyaluronidase pretreatment	[71]
anti-PSMA		B6.Cg-Foxn1 nu/J and C57BL/6J mice	IM + EP	[75]
anti-CD4, anti-influenza, anti-Ebola		BALB/c mice	IM + EP, IM + EP with hyaluronidase pretx	[146]
anti-Zaire ebolavirus glycoprotein	modification of N terminus amino acids back to germline	BALB/c (anti-CD4 and anti-CD8 transient depletion)	IM + EP with hyaluronidase	[68]
anti-CTLA-4	Sequence modifications based on sequence alignment to the mouse germline sequence	C57Bl/6, BALB/c (anti-CD4 and anti-CD8 transient depletion)	IM + EP with hyaluronidase	[147]
anti-HER2		BALB/c, athymic nude, RAG2−/−gc−/−	IM + EP with hyaluronidase pretx	[73]
anti-ZIKV E protein DIII domain (DMAb-ZK190)	LALA mutation	C57BL/6 mice, Rhesus macaques	IM + EP with hyaluronidase pretreatment in mice, IM + EP only in rhesus macaques	[77]
anti-OspA Lyme	framework modification of the WT variant	C3H mice	IM + EP with hyaluronidase	[148]
anti-PCSK9		C57BL/6J wild-type and nude B6.Cg-foxn1nu/J mice	IM + EP	[149]
anti-HER2		Nu/J mice	IM + EP with hyaluronidase	[78]
anti-PD-1		BALB/c mice	IM + EP	[150]
anti-human CEA, anti-human EGFR, anti-HER2		Swifter sheep, C57BL/6J RAG1 ko mice	IM + EP with hyaluronidase pretx	[151]
multiple HIV-1-specific bNAbs	modification of the C- and N-terminus of the variable region to germline	BALB/c (anti-CD4 and anti-CD8 transient depletion), Rhesus macaques	IM + EP with hyaluronidase	[79]
anti-HBV		athymic nude CAnN.Cg-Foxn1nu/Crl mice	IM + EP	[152]
anti-mCTLA-4 (9D9), anti-ratPD-1		C57BL/6J mice	IM + EP with hyaluronidase pretx	[153]
			Intratumoral + EP	
anti-ZIKV envelope		B6.Cg-Foxn1nu/J mice	IM + EP	[154]
2C7, directed against a lipooligosaccharide glycan epitope, Neisseria gonorrhoeae	two complement enhancing variants, HC_E430G and HC_E345K, one complement abrogating variant HC_K322A/D270A	Jh mice, nude mice	IM + EP with hyaluronidase	[155]
anti-HER2		BALB/c mice	IM + EP with hyaluronidase pretx	[156]
anti-SARS-CoV2 spike	L234F/L235E/P331S; “TM” to ablate FcR and C1q binding, M252Y/S254T/T256E; “YTE” to promote FcRn-mediated recycling	BALB/c	IM + EP with hyaluronidase	[70]
mAb clones CIS43, 317, and L9, which target a junctional epitope, major repeat, and minor repeat of the Plasmodium falciparum circumsporozoite protein (CSP), respectively	reverting specific, non-essential residues in the framework region back to germline configuration	BALB/cJ (anti-CD4 and anti-CD8 transient depletion)	IM + EP with hyaluronidase	[69]
anti-human CEA		Swifter sheep	IM + EP with hyaluronidase	[157]
**mRNA-Based delivery**				
**Encoded mAb(s)**	**Modifications**	**Experimental Model**	**Mode of Delivery**	**Reference**
anti-CD3/anti-claudin 6 (CLDN6), anti-CD3/anti-caludin 18.2 (CLDN18.2), anti-CD3/anti-epithelial cell adhesion molecule (EpCAM), anti-CD3/(anti-CLDN6)2	1-methylpseudouridine	NOD.Cg-Prkdscid IL2rgtm1Wjl/SzJ (NSG) mice	Formulation with TransIT-mRNA Transfection kit, IV	[99]
anti-HIV Env (VRC01)	1-methylpseudouridine	BALB/c mice	LNP, IV	[97]
anti-Rabies glycoprotein G, anti-Botulinum neurotoxin serotype A (VHH-based neutralizing agent, VNA), anti-CD20, anti-HIV gp120, anti-Influenza B HA, anti-Shiga toxin 2 (VNA)	two fused VHHs complemented by an albumin-binding peptide	Swiss-Albino mice (rabies and influenza b) CD1 mice (VNAs)	LNP, IV	[100]
anti-Influenza A	1-methylpseudouridine	cynomolgus monkeys	LNP, IV	[98]
anti-CHIKV E2 glycoprotein (CHIKV-24)		cynomolgus monkeys	LNP, IV	[95]
anti-HER2		C57BL/6 mice	cKK-E12 (also known as MD-1) lipid-like nanoparticles, IV	[127]
Anti-ZIKV Env (ZIKV-117)	alphavirus replicon (replicating viral RNA that amplifies) 1-methylpseudouridine	C57BL/6 mice	Nanostructured lipid carrier, IM	[129]
anti-HIV GP120 (PGT121)	1-methylpseudouridine	female Katahdin ewes	aerosol delivery of unformulated mRNA in water	[96]
anti-Influenza A matrix protein 2/anti-mouse Fcγ receptor IV	1-methylpseudouridine		DOTAP (1,2-dioleoyl-3-trimethylammonium-propane)/cholesterol nanoparticles delivered intratracheally	[158]
anti-CHIKV Env (mRNA-1944)		human	LNP, IV	[126]
Poxviruses: Mature virion, c7D11 (anti-L1); Enveloped virion, c8A (anti-B5) and c6C (anti-A33)		New Zealand White rabbits	LNP, IM jet injection	[159]
anti-PD-1	1-methylpseudouridine	C57BL/6 mice	LNP, IV	[128]
anti-SARS-CoV-2	LS mutation (M428L/N434S) and GPI anchor 1-methylpseudouridine	Golden Syrian hamster	poly-beta amino thio ester (PBATE), nebulizer	[133]

## 6. Summary and Conclusions

Monoclonal antibodies (mAbs) have demonstrated remarkable efficacy, offering target specificity and high affinity. However, their widespread use remains limited, especially in low- and middle-income countries. Gene-based approaches have emerged as promising alternatives to current synthetic production methods for mAbs to address this challenge. These approaches involve delivering nucleic acid encoding the desired mAb, which can significantly reduce production costs, resource requirements, and developmental time compared to protein-based mAb therapies. Moreover, gene-based strategies enable sustained delivery and in vivo generation of mAbs with proper post-translational modifications, eliminating the need for repeated infusions or injections. The implementation of gene-based approaches has the potential to enhance the accessibility and affordability of mAb therapies, making them more available to patients worldwide.

Furthermore, gene-based modalities such as viral vectors, DNA, and mRNA offer distinct advantages for mAb therapy (Table 1). mRNA-based mAb therapy provides immediate and transient expression, making it suitable for transient infectious diseases where prolonged mAb exposure is unnecessary. On the other hand, sustained mAb production, crucial for cancer or chronic infections, can be achieved using AAV- or DNA-based approaches. AAV is preferred for high mAb concentrations, while DNA delivery offers self-limited but repeatable administration, potentially increasing safety. Additionally, the advancement of nucleic acid technology has led to the exploration of circular RNAs, which exhibit enhanced stability compared to linear RNAs due to their resistance to exonucleases. This stability allows for higher and more durable protein expression. The use of nucleic acid-encoded mAb treatments holds tremendous promise in improving access to life-saving mAbs. Given their safety, efficacy, and scalability, translating nucleic acid technology into clinical practice and the routine use of nucleic acid-encoded mAbs in healthcare settings are highly likely.

## 7. Future Perspectives

The success of nucleic acid-based approaches, exemplified by the effective SARS-CoV-2 vaccines against COVID-19, has paved the way for their increasing utilization in the near future. These approaches offer advantages such as facile synthesis and expedited development and production scale-up, positioning them as a preferred platform for vaccine and therapeutic delivery. However, the use of an adeno-associated virus (AAV) as a delivery system may be hampered by pre-existing or acquired immunity to the vector, which would limit the ability for repeat dosing. DNA-based delivery platforms do not induce significant anti-vector responses and thus can be administered multiple times to the same patient. However, efficient in vivo uptake and expression of transgenes from DNA plasmids requires the use of electroporation-enhanced delivery, which poses challenges such as electroporation-induced muscle contractions and tissue damage. Additionally, electroporation machines are specialized equipment that are not commonly available and would require additional training for medical personnel before they can be used (Table 2).

Conversely, mRNA and circular RNA-encoded monoclonal antibodies (mAbs) hold significant promise as relatively novel therapeutics due to their favorable safety profiles and repeatable dosing. Nevertheless, the development of lipid nanoparticle (LNP) formulations for mRNA delivery requires meticulous formulation to mitigate immune-mediated and cellular toxicities. Despite these obstacles, encouraging results from clinical trials underscore the need for further refinement and expansion of nucleic acid-based approaches. This burgeoning area of research bears tremendous potential, offering cost-effective medicines facilitated by nucleic acid-based mAbs.

The antibody is selected based on its binding capacity and functionality, such as neutralization, agonist/antagonist activity, or antibody-dependent cellular cytotoxicity. Next, the antibody’s VH and VL chain sequences are obtained and optimized to create a mAbs-encoding gene cassette. This cassette can then be used to create antibody-encoding (i) mRNA by in vitro transcription (IVT) using a DNA template (plasmid or PCR product); the transcribed mRNA is then formulated into lipid nanoparticles (LNP) and delivered into skeletal muscle cells via intramuscular (i.m.) injection; (ii) AAV particles by cloning the cassette into AAV genome construct and then transfecting this into a packaging cell line with a helper virus; recovered viruses are purified and concentrated and can be delivered into skeletal muscle cells via i.m injection; or (iii) DNA by cloning it into a DNA plasmid downstream of a strong eukaryotic promoter. The plasmid can be delivered into skeletal muscle cells via electroporation-enhanced i.m. injection. The muscle cells then transcribe and/or translate the delivered antibody gene cassette and produce antibodies that are secreted into the bloodstream and circulate throughout the body. Alternatively, the mRNA can be encapsulated in liver-targeting LNPs and delivered via intravenous (i.v.) injection, where it is transported through the bloodstream to the liver. In the liver, the protein (Abs) is synthesized in hepatocytes. The LS leader sequence is used to optimize protein secretion. VH represents the variable heavy chain, FC is the furin cleavage site, and VL stands for the variable light chain.

## Figures and Tables

**Table 2:** Comprehensive table to summarize the different modes of delivery.

Mode of mAb Delivery	Advantages	Disadvantages
Adeno-Associated Virus (AAV)	(1) High serum concentrations	(1) Vector immunity (2) Risk of insertional mutagenesis (3) Insert capacity limitation (4) Time to peak expression can take months (5) Expensive and complex manufacturing (6) Cold chain requirement
Deoxyribonucleic Acid (DNA)	(1) Repeatable dosing (2) Easily manufactured and manipulated (3) Stable at room temperature	(1) Delivery oftentimes requires electroporation (2) Time to peak expression can take weeks
Messenger RNA (mRNA)	(1) Repeatable dosing (2) Only a few hours for expression with peak expression within days (3) Cell-free production	(1) LNP toxicity (2) High cost for manufacturing and upscaling (3) Instability during long-term storage (4) Cold chain requirement

## Data Availability

Not. applicable.
